# Supplementary material for: The Current State of Mock Circulatory Loop Applications in Aortic and Cardiovascular Research: A Scoping Review
Source: Biomedicines. 2025 Dec 22;14(1):28. doi: 10.3390/biomedicines14010028 (PMC12839285; doi:10.3390/biomedicines14010028)
Supplement: Supplementary file 1 [file biomedicines-14-00028-s001.zip › biomedicines-4001562-supplementary.pdf]

Supplementary Materials

Table S1. Search String for PUBMED, Web of Science and Scopus, used for a scoping review on mock circulatory loops in aortic research.

**Pubmed**	("mock circulatory loop"[Title/Abstract] OR "mock circulation loop"[Title/Abstract] OR MCL[Title/Abstract] OR "ex vivo"[Title/Abstract] OR "bench model"[Title/Abstract] OR phantom[Title/Abstract])<br>AND<br>(aorta[Title/Abstract] OR aortic[Title/Abstract])<br>AND<br>("stent graft"[Title/Abstract] OR stent[Title/Abstract] OR TEVAR[Title/Abstract] OR endovascular[Title/Abstract] OR repair[Title/Abstract] OR "pulse wave velocity"[Title/Abstract] OR "aortic stiffness"[Title/Abstract] OR "radial strain"[Title/Abstract])<br>AND<br>(model[Title/Abstract] OR phantom[Title/Abstract] OR synthetic[Title/Abstract] OR biological[Title/Abstract] OR porcine[Title/Abstract] OR human[Title/Abstract] OR cadaveric[Title/Abstract] OR preserved[Title/Abstract] OR fresh[Title/Abstract])	**338**
**Web of Science**	TS=("mock circulatory loop" OR "mock circulation loop" OR MCL OR "ex vivo" OR "bench model" OR phantom) AND TS=(aorta OR aortic) AND TS=("stent graft" OR stent OR TEVAR OR endovascular OR repair OR "pulse wave velocity" OR "aortic stiffness" OR "radial strain") AND TS=(model OR phantom OR synthetic OR biological OR porcine OR human OR cadaveric OR preserved OR fresh)	**991**
**Scopus**	TITLE-ABS-KEY("mock circulatory loop" OR "mock circulation loop" OR MCL OR "ex vivo" OR "bench model" OR phantom)<br>AND<br>TITLE-ABS-KEY(aorta OR aortic)<br>AND<br>TITLE-ABS-KEY("stent graft" OR stent OR TEVAR OR endovascular OR repair OR "pulse wave velocity" OR "aortic stiffness" OR "radial strain")<br>AND<br>TITLE-ABS-KEY(model OR phantom OR synthetic OR biological OR porcine OR human OR cadaveric OR preserved OR fresh)	**734**

Table S2. Study characteristics of all included studies included in a scoping review on mock circulatory loops in aortic research. *PWV: pulse wave velocity; TEVAR: thoracic endovascular aortic repair; PIV: particle image velocimetry; EVHP: Ex-vivo heart perfusion; TL: true lumen; FL: false lumen; SPCCT: spectral photon-counting computed tomography; ILT: intraluminal thrombus; AAA: abdominal aortic aneurysm; MRI: magnetic resonance imaging; PWI: pulse wave imaging; REBOA: resuscitative endovascular balloon occlusion of the aorta; MAP: mean arterial pressure; LT: Limb thrombosis; EVAR: endovascular aortic repair; IVUS: intravascular ultrasound; FSI: fluid-structure interaction; CFD: computational fluid dynamics CMR: cardiovascular magnetic resonance; FDR: flow division ratio; WSS: wall shear stress; SNR: Signal-to-noise ratio; CNR: contrast-to-noise ratio.*

First Author	*Year*	*Theme*	*Primary Outcome*	*Measured Unit*	*Flow Type*	*Pump Type*	*Working Fluid*	*Aorta Type*	*Patient-Specific*	*N Aortic Samples*	*Abdominal / Thoracic Environment*
Agrafiotis et al.	2023	(Bio)mechanical characteristics, Intervention	The effect of stent-graft implantation on aortic compliance and stiffness.	Distensibility (%), stress–stretch (kPa), Δd_circ (mm), pressure (mmHg)	Pulsatile	Piston Pump	Glycerol/ glycerin	Human	No	9	Immersed in 37 °C glycerin bath.
Amabili et al.	2020	(Bio)mechanical characteristics, No intervention, Healthy	The viscoelastic material parameters for the human descending thoracic aorta	Storage modulus (kPa) and Loss factor (dimensionless).	Pulsatile	Piston Pump	Glycerol/ glycerin	Human	No	11	-
Amibili et al.	2020	Devices/Products, Surgical	Viscoelastic properties of Dacron aortic grafts under physiological flow.	Storage modulus (MPa), Loss factor (dimensionless), and cyclic diameter change (%).	Pulsatile	Piston Pump	Glycerol/ glycerin	Graft only	No	2	-
Bianchi et al.	2022	(Bio)mechanical characteristics, Intervention	Impact of TEVAR on aortic stiffness and local wall stress.	PWV (m/s), von Mises stress (MPa), strain (%)	Pulsatile	Piston Pump	Water-based	Porcine	No	5	-
Birjiniuk et al.	2017	Hemodynamics, No intervention, Pathological	Hemodynamic changes in the aorta as a result of a dissection.	Reverse Flow Index (%), flow rate (L/min or mL/s), velocity (cm/s), flap displacement (mm).	Pulsatile	Unknown	Water-based	Phantom (mold)	Yes	-	-
Blackwood et al.	2016	Hemodynamics, Intervention	Aneurysm sac pressurization and endoleak visibility in a model of endotension.	Pressure (mmHg), flow (mL/min), visualization time (sec/min)	Pulsatile	Unknown	Water-based	Phantom (3D printed)	Yes	1	-
Boese et al.	2000	(Bio)mechanical characteristics, No intervention, Healthy	Estimation of aortic compliance.	PWV (m/s), compliance (mm²/kPa) indirectly	Pulsatile	Continuous Pump + Valve	Water-based	Synthetic Tube	No	1	-
Büsen et al.	2017	Hemodynamics, No intervention, Healthy	Effect of aortic compliance on flow patterns and pressure dynamics in an in vitro aortic arch model measured by PIV.	Compliance ($\times 10^{-3}$ $mmHg^{-1}$), pressure change (dp/dt) (mmHg/s), velocity (cm/s), flow (L/min), area (cm^2)	Pulsatile	Indirect Compression System	Glycerol/ glycerin	Phantom (3D printed)	Yes	1	-
Cameron et al.	2020	(Bio)mechanical characteristics, No intervention, Healthy	Impact of mock aortic compliance on pump energy output, pressure waveform shape, and downstream flow	Distensibility ($\times 10^{-3}$ $mmHg^{-1}$), pressure (mmHg), velocity (m/s), energy output (J/min), flow (L/min)	Pulsatile	Indirect Compression System	Water-based	Phantom (mold)	No	1	-

			steadiness in an EVHP system								
Canaud et al.	2008	Devices/Products, Surgical	Lack of device–wall apposition of thoracic stent-grafts as a function of oversizing and aortic arch angulation	Lack of apposition (mm), angulation (°), oversizing (%), pressure (mmHg)	Pulsatile	Diaphragm / sac-type Pump	Glycerol/ glycerin	Human	No	15	-
Canchi et al.	2018	Hemodynamics, No intervention, Pathological	Quantification of the effect of distal re-entry tears on lumen pressure differential, flap strain, and vessel morphology in type B aortic dissection using an ex vivo porcine model and clinical comparison.	Pressure difference (mmHg), FDR (dimensionless), circumferential flap strain (%), flow rate (L/min), pulse pressure (mmHg).	Pulsatile	Piston Pump	Blood Mimicking	Porcine	No	16	-
Chen et al.	2022	Hemodynamics, No intervention, Pathological	Flow distribution ratio, pressures in TL/FL and branches, velocity patterns	Flow (L/min, % FDR), pressure (mmHg), velocity (cm/s)	Pulsatile	Gear Pump	Glycerol/ glycerin	Phantom (mold)	Yes	1 normal phantom, 1 AD phantom	-
Chung et al.	2000	Hemodynamics, Intervention	Presence/absence of TL collapse and branch perfusion compromise	Collapse status (LC, TB all, LE), branch flow rates, lumen pressures	Pulsatile	Diaphragm / sac-type Pump	Water-based	Synthetic Tube	No	2 phantoms (compliant + rigid)	-
Chung et al.	2000	Hemodynamics, No intervention, Pathological	Relief or prevention of TL collapse (branch perfusion recovery)	Qualitative collapse status (CR, LC, LE, TB all), flow restoration	Pulsatile	Diaphragm / sac-type Pump	Water-based	Synthetic Tube	No	2 phantoms (compliant + rigid)	-
Cosset et al.	2023	Diagnostics	Feasibility of bicolor K-edge SPCCT imaging to identify and characterize thoracic endoleaks in a dynamic phantom	Contrast concentration (mg/mL), CT attenuation (HU), flow rate (L/min)	Continu ous	Centrifugal Pump	Water-based	Phantom (3D printed)	Yes	1	-
de Beaufort et al.	2017	(Bio)mechanical characteristics, Intervention	Change in aortic stiffness after TEVAR, quantified by PWV and pulse pressure.	PWV (m/s), pulse pressure (mmHg), flow (L/min), stent coverage (%)	Pulsatile	Piston Pump	Water-based	Porcine	No	15	-
de Beaufort et al.	2017	(Bio)mechanical characteristics, Intervention	Change in aortic stiffness after stent-graft deployment and extension, expressed as PWV.	PWV (m/s), pressure (mmHg), flow (L/min), stent coverage length (mm)	Pulsatile	Piston Pump	Water-based	Porcine	No	20	-
de Hoop et al.	2020	Diagnostics	Improvement in motion tracking and strain estimation accuracy of the abdominal aorta using multiperspective ultrasound imaging.	Strain (%), displacement (mm), pressure (mmHg), flow (L/min), heart rate (bpm).	Pulsatile	Piston Pump	Saline-buffered	Porcine	No	3	A 3D printed spine and gelatin solution were added to mimic the surrounding tissues in the abdomen.
de Hoop et al.	2025	Diagnostics	Accuracy improvement of 3-D vascular motion tracking and strain imaging in ex vivo porcine aortas using bistatic dual-	Displacement (mm), strain (%), pressure (mmHg), flow (L/min), signal-to-noise ratio (dB	Pulsatile	Gear Pump	Water-based	Porcine	No	3	A 3D printed spine and gelatin solution were added to mimic the surrounding tissues in the abdomen.

			aperture ultrasound acquisition								
de Kort et al.	2024	(Bio)mechanical characteristics, Intervention	Change in aortic PWV after open surgical interposition grafting of the proximal descending aorta in an ex vivo porcine model	PWV (m/s), blood pressure (mmHg), flow (L/min)	Pulsatile	Piston Pump	Water-based	Porcine	No	15	-
Desai et al.	2011	Devices/Products, Phantom , Healthy	Development and validation of a compliant nanocomposite aortic model replicating physiologic viscoelasticity for stent-graft testing	Compliance (%/mmHg × 10^{-2}), stiffness index (b), pressure (mmHg), flow (mL/min)	Pulsatile	Centrifugal Pump	anticoagulated blood	Phantom + porcine	No	6	Immersed in saline maintained at 37C.
Dziodzio et al.	2010	Hemodynamics, No intervention, Pathological	Influence of primary entry tear location on antegrade and retrograde propagation in acute type B aortic dissection.	Pressure (mmHg), flow (L/min), propagation length (mm), time (min).	Pulsatile	Diaphragm / sac-type Pump	Unknown	Porcine	No	26	-
Ene et al.	2011	(Bio)mechanical characteristics, No intervention, Pathological	Effect of ILT presence, stiffness, and thickness on AAA wall dynamics under pulsatile flow.	Compliance ($\times 10^{-4}$/mmHg), pressure (mmHg), diametral strain (%), elastic modulus (MPa), flow rate (L/min).	Pulsatile	Piston Pump	Glycerol/ glycerin	Phantom (mold)	No	4	-
Faure et al.	2014	Hemodynamics, No intervention, Pathological	Reproducibility of a human ex vivo type B aortic dissection model and analysis of dissection propagation according to the primary entry tear location	Pressure (mmHg), propagation length (% reaching celiac, renal, or infrarenal aorta), flow rate (L/min), heart rate (bpm).	Pulsatile	Diaphragm / sac-type Pump	Glycerol/ glycerin	Human	No	20	-
Ferrari et al.	2019	Devices/Products, Surgical	Characterization of the dynamic mechanical response of Dacron aortic grafts under pulsatile flow in a mock circulation loop.	Displacements (diameter change, bending), pressure (mmHg), flow (L/min).	Pulsatile	Piston Pump	Saline-buffered	graft only	No	1	-
Franchini et al.	2022	(Bio)mechanical characteristics, No intervention, Healthy	Quantification of viscoelastic properties of the human descending thoracic aorta under pulsatile loading using a mock circulatory loop.	Pressure (mmHg), diameter change (mm/%), flow (L/min), η (dimensionless), dynamic stiffness ratio (dimensionless), density (g/cm^3	Pulsatile and quasi-static	Piston Pump	Glycerol/ glycerin	Human	No	2	-
Gaddum et al.	2014	(Bio)mechanical characteristics, No intervention, Healthy	Quantification of beat-to-beat variation in PWV during respiration using real-time MRI.	Pulse wave velocity (m/s), heart rate (bpm), pressure (mmHg).	Pulsatile	Piston Pump	Unknown	Mechanical	No	1	-
Hauck et al.	2021	Devices/Products, Surgical	Evaluation of deployment accuracy of a novel ascending aortic stent-	Deployment deviation (mm), distance from landing zone (mm),	Unknown	Unknown	Unknown	Phantom (3D printed)	Yes	3	-

			graft design in phantom, cadaver, and clinical application.	pressure (mmHg), flow rate (L/min).							
Jansen et al.	2020	Devices/Products, Surgical	Assessment of safety, accuracy, and feasibility of Fiber Optic RealShape (FORS) technology for real-time 3-dimensional endovascular navigation.	Cannulation success rate (%), tip-to-tip accuracy (mm), lateral offset (mm), radiation exposure (mSv).	Continu ous	Unknown	Water-based	Phantom (mold)	No	1	-
Jarman et al.	2025	(Bio)mechanical characteristics, No intervention, Healthy	Comparison of different indices (strain, compliance, distensibility, PWV, stiffness indices) to quantify aortic elasticity under variable pressure conditions	Pressure (mmHg), PWV (m/s), strain (%), distensibility ($\times10^{-3}$ $mmHg^{-1}$), compliance (mm/mmHg)	Pulsatile	Piston Pump	Saline-buffered	Porcine + Ovine	No	6 ovine samples, 6 porcine samples	-
Kratzberg et al.	2009	Devices/Products, Surgical	Effect of graft oversizing on fixation strength and barb penetration in barbed endovascular grafts.	Oversizing (%), pullout force (N), barb penetration depth (% of barb length), pressure (mmHg), flow rate (L/min)	Pulsatile	Piston Pump	Saline-buffered	Bovine + Phantom	No	-	-
Lan et al.	2024	Devices/Products, Radiological	Validation of the Reduced Unified Continuum Formulation (RUCF) for predicting aortic hemodynamics against in vitro 4-dimensional Flow MRI measurements.	Pressure (mmHg), Flow (mL/s), Velocity (cm/s), Lumen area (relative change), PWV (cm/s)	Pulsatile	Gear Pump	Glycerol/ glycerin	Phantom (3D printed)	Yes	1	The 3D-printed aortic flow phantom was embedded in a gel block to ensure repeatable positioning and to provide a static ‘‘tissue’’ reference for eddycurrent phase offset correction.
Legerer et al.	2020	(Bio)mechanical characteristics, Intervention	Experimental evaluation of a novel prosthetic aortic design concept to reduce turbulence and improve flow distribution.	Flow velocity (m/s), pressure drop (mmHg), Reynolds number (–), wall shear stress (Pa).	Pulsatile	Indirect Compression System	Glycerol/ glycerin	graft only	No	1	-
Li et al.	2015	Diagnostics	Performance assessment of PWI to optimize spatial and temporal parameters for accurate PWV measurement.	PWV (m/s), correlation coefficient (r^2), frame rate (fps), displacement (mm).	Pulsatile	Roller (Peritaltic) Pump	Saline-buffered	Canine	No	2	-
Madurska et al.	2020	Devices/Products, Surgical	Comparison of compliant versus semi-compliant balloon systems for REBOA and evaluation of a safety-valve mechanism to prevent over-inflation injury.	Pressure (atm/mmHg), balloon volume (mL), working length (mm), circumferential stretch ratio (–).	Continu ous	Unknown	Saline-buffered	Porcine	No	12	-
Maleckis et a.	2021	Devices/Products, Surgical	Determination of safe balloon inflation parameters for REBOA in	Inflation pressure (mmHg), inflation volume (mL), rupture pressure	Pulsatile	Piston Pump	Saline-buffered	Human	No	79	-

			ex vivo human aortas under varying flow pressures.	(mmHg), flow pressure (mmHg).							
Mandigers et al.	2024	(Bio)mechanical characteristics, No intervention, Healthy	Effect of type III aortic arch angulation on aortic stiffness and flow dynamics following stent-graft implantation	PWV (m/s), pressure (mmHg), flow (L/min), arch angle (°)	Pulsatile	Piston Pump	Water-based	Porcine	No	24; 15 with a stent-graft	-
Mandigers et al.	2023	(Bio)mechanical characteristics, Intervention	Difference in post-deployment aortic stiffness and pulse wave velocity between two generations of thoracic stent-grafts in an ex vivo porcine model.	PWV (m/s), Pulse Pressure (mmHg), Flow (L/min)	Pulsatile	Piston Pump	Water-based	Porcine	No	20 (10 Captivia, 10 Navion)	-
Mascarenhas et al.	2016	(Bio)mechanical characteristics, No intervention, Healthy	Quantification of aortic wall elasticity under physiological loading using 2-dimensional ultrasound elastography	Shear modulus (kPa), Stress–strain curves, Diameter change (mm)	Pulsatile	Piston Pump	Saline-buffered	Porcine	No	12	-
McCarthy et al.	2019	Devices/Products, Surgical	Ability to maintain a user-defined proximal MAP setpoint via automated balloon control	Pressures (mmHg), MAP, systolic, diastolic	Pulsatile	Unknown	Unknown	Synthetic Tube	No	1	-
Meess et al.	2017	Training, Surgical	Simulation fidelity, procedural deviations, impact on clinical planning	Procedural step deviations (impact scoring), fluoroscopic imaging comparisons	Pulsatile	Piston Pump	Unknown	Phantom (3D printed)	Yes	1	-
Mirgolbabaee et al.	2023	Hemodynamics, Intervention	Identification of hemodynamic predictors of LT after EVAR with the Anaconda endograft using ultrasound particle image velocimetry	VC (0–1), RT (s), velocity (cm/s)	Pulsatile	Piston Pump	Glycerol/ glycerin	Phantom (3D printed)	Yes	1	-
Mirgolbabaee et al.	2025	Devices/Products, Phantom	Quality of averaged AAA model (Sørensen–Dice similarity), acoustic parameters, feasibility of echoPIV	Sørensen–Dice coefficient, SoS (m/s), attenuation (dB/mm), velocity (cm/s), volumetric flow (mL/s)	Pulsatile	Piston Pump	Glycerol/ glycerin	Phantom (3D printed)	No	1	-
Mohl et al.	2024	Devices/Products, Phantom	Feasibility of TEVAR in phantom, physiological flow/pressures, compatibility with imaging	Flow (L/min), velocity (cm/s), pressure (mmHg), imaging visibility	Pulsatile	Piston Pump	Glycerol/ glycerin	Phantom (3D printed)	Yes	1	-
Moravia et al.	2022	(Bio)mechanical characteristics, No intervention, Healthy	Validation of the lnD–U method for estimating PWV and Young's modulus in an aorta phantom using PIV.	PWV (m/s), Young's modulus (MPa), Diameter change (mm)	Pulsatile	Unknown	Glycerol/ glycerin	Phantom (3D printed)	Yes	1	-

Moresco et al.	2000	Diagnostics	Vessel diameter differences between CO2, iodinated contrast, and IVUS.	Vessel diameter (mm)	Pulsatile	Roller (Peritaltic) Pump	Glycerol/ glycerin	Phantom (mold)	No	1	-
Morris et al.	2013	Devices/Products, Surgical	Influence of stent-graft compliance and bifurcation geometry on hemodynamics and wave reflection in AAA models	Compliance (10^{-4}/mmHg), PWV (m/s), reflection coefficient (%), WSS (Pa), systolic pressure (mmHg)	Pulsatile	Piston Pump	Glycerol/ glycerin	Phantom (3D printed)	Yes	1 silicone phantom + 2 rapid prototyped rigid bifurcation models	-
Morris et al.	2016	(Bio)mechanical characteristics, Intervention	Quantification of device/arterial wall compliance mismatch for five AAA devices under physiological flow.	Compliance (10^{-4}/mmHg), PWV (m/s), PAEL (mm/mmHg), reflection coefficient (%)	Pulsatile	Piston Pump	Glycerol/ glycerin	Phantom (3D printed)	Yes	1 phantom	-
Morris et al.	2022	Hemodynamics, No intervention, Pathological	Assessment of hemodynamic features within true and false lumens in a patient-specific type B aortic dissection model	Pressures (mmHg), velocities (cm/s), flap displacement (mm), % branch flow	Pulsatile	Piston Pump	Blood Mimicking	Phantom (3D printed)	Yes	1	-
Nagel et al.	2025	Devices/Products, Radiological	Determination of the minimal tantalum concentration in the AneuFix polymer required for sufficient detectability during fluoroscopy in type II endoleak treatment.	Tantalum concentration (%), contrast-to-noise ratio (CNR), detection distance (mm), flow rate (mL/min), pressure (mmHg)	Pulsatile	Roller (Peritaltic) Pump	Water-based	Phantom (3D printed)	No	10 different static phantoms, 3 of each phantom	The endoleak phantom was placed inside a customized phantom of the abdomen, featuring tissue-mimicking material, kidneys, part of the liver and spleen, to provide a realistic background during fluoroscopy and CT.
Nair et al.	2025	Hemodynamics, No intervention, Pathological	Assessment of exercise-induced pressure drop across aortic coarctations using hybrid mock circulation loop and FSI simulations.	Pressure drop (mmHg), cardiac output (L/min), heart rate (bpm), flow (mL/s), systemic vascular resistance (mmHg·s/mL)	Pulsatile	Indirect Compression System	Glycerol/ glycerin	Phantom (3D printed)	Yes	5	-
Nauta et al.	2016	(Bio)mechanical characteristics, Intervention	Change in longitudinal aortic strain before and after TEVAR under physiological pressure conditions	Longitudinal strain (%), tensile strength (MPa)	Continu ous	Centrifugal Pump	Water-based	Porcine	No	20	-
Nauta et al.	2017	(Bio)mechanical characteristics, Intervention	Change in radial aortic strain before and after TEVAR under physiological pressure conditions	Radial strain (%), Young's modulus (MPa)	Continu ous	Centrifugal Pump	Water-based	Porcine	No	20	-

Ong et al.	2019	Hemodynamics, Intervention	Evaluation of hemodynamic performance and wall shear effects of a novel slit-perforated stent-graft design for thoracic aortic aneurysms using CFD and experimental validation.	Flow velocity (m/s), wall shear stress (Pa), pressure (mmHg), flow rate (L/min).	Pulsatile	Piston Pump	Glycerol/ glycerin	Phantom (3D printed)	No	3	-
Pasta et al.	2016	Hemodynamics, Intervention	Quantification of stent-graft displacement and rotation in relation to flow and pressure for varying bird-beak configurations in aortic arch phantoms.	Displacement (mm), rotation (°), pressure (mmHg), flow (L/min), pulse pressure (mmHg).	Pulsatile	Piston Pump	Glycerol/ glycerin	Phantom (3D printed)	Yes	1	-
Peelukhana et al.	2017	Hemodynamics, No intervention, Pathological	Determination of the influence of pulse pressure and entry tear geometry on propagation of acute type B aortic dissections.	Pulse pressure (mmHg), propagation length (cm), true lumen area (%), de-cohesive energy (J/m²).	Pulsatile	Piston Pump	Blood Mimicking	Porcine	No	36	-
Perrot et al.	2017	Devices/Products, Phantom	Validation of a biofidelic abdominal aorta phantom combining ultrasound and digital image stereo-correlation for realistic EVAR simulation.	Displacement (mm), strain (%), pressure (mmHg), flow rate (L/min).	Pulsatile	Piston Pump	Unknown	Phantom (3D printed)	Yes	1	A patient-specific silicone phantom was mounted on a 3D-printed curved spine with springs to reproduce vascular tortuosity and mechanical support from surrounding tissues. Abdominal pressure could be mimicked by immersing the phantom in water, which was necessary for ultrasound acquisitions but not for DISC measurements.
Potthast et al.	2007	Diagnostics	Comparison of intra-arterial contrast-enhanced MR aortography with and without parallel acquisition for imaging quality and diagnostic accuracy.	SNR, CNR, acquisition time (s), spatial resolution (mm).	Pulsatile	Unknown	Saline-buffered	Synthetic Tube	No	1	-
Qing et al.	2016	Hemodynamics, Intervention	Assessment of persistent intraluminal pressure in the false lumen after endovascular stent grafting for type B aortic dissection.	Pressure (mmHg), pressure index (% of systemic pressure), flow rate (mL/s), velocity (cm/s).	Pulsatile	Piston Pump	Other	Porcine	No	28 (A=10, B=12, C=6)	-

Rengier et al.	2012	Devices/Products, Radiological	Evaluation of the effect of a nitinol stent-graft on phase-contrast MRI flow measurements in the thoracic aorta.	Flow rate (mL/s), velocity (cm/s), SNR, percentage deviation (%).	Pulsatile	Gear Pump	Glycerol/ glycerin	Synthetic Tube	No	`1	-
Riga et al.	2011	Devices/Products, Radiological	Evaluation of robotic catheter performance versus conventional methods during fenestrated stent grafting in an aneurysm phantom.	Procedure time (min), number of catheter movements, IC3ST performance score (/35), radiation exposure (mSv).	Pulsatile	Unknown	Glycerol/ glycerin	Phantom (3D printed)	No	2	-
Riga et al.	2010	Devices/Products, Radiological	Assessment of robotic versus manual catheter performance for arch vessel cannulation under pulsatile flow.	Cannulation time (min), number of catheter movements, vessel wall hits (n), IC3ST score (/35).	Pulsatile	Unknown	Glycerol/ glycerin	Phantom (3D printed)	No	1	-
Roberts et al.	2015	Diagnostics, Radiological, Healthy	Validation of real-time CMR for PWV measurement during exercise stress testing.	Pulse wave velocity (m/s), heart rate (bpm), mean arterial pressure (mmHg), cardiac output (L/min).	Pulsatile	Piston Pump	Unknown	Synthetic Tube	No	2	-
Rudenick et al.	2013	Hemodynamics, No intervention, Pathological	Evaluation of the influence of tear size and configuration on true and false lumen hemodynamics in chronic type B aortic dissection.	Pressure (mmHg), velocity (cm/s), flow rate (L/min), false lumen pressure index (% of TL pressure).	Pulsatile	Piston Pump	Water-based	Phantom (mold)	No	8	-
Saida et al.	2013	Diagnostics	Visualization and detection of endoleaks after EVAR using noninvasive flow-sensitive 4D phase-contrast MRI.	Flow velocity (cm/s), pressure (mmHg), signal intensity ratio (%), flow volume (mL/s).	Pulsatile	Indirect Compression System	Saline-buffered	Phantom (3D printed)	No	4	The aortic aneurysm model was sunk into an acrylic pool filled with saline. Several bottles filled with copper sulfate solution were also sunk to improve magnetic field inhomogeneity.
Schellinger et al.	2023	(Bio)mechanical characteristics, Intervention	Quantification of vascular stiffness gradients and PWV changes immediately after endograft implantation in an ex vivo porcine model.	PWV (m/s), strain (%), pressure (mmHg).	Pulsatile	Diaphragm / sac-type Pump	Saline-buffered	Porcine	No	20 (10 stented, 10 controls)	-
Schurink et al.	1998	Hemodynamics, Intervention	Assessment of aneurysmal sac pressure and imaging detectability under varying endoleak diameters after endovascular repair.	Pressure (mmHg), equilibration time (min), endoleak diameter (mm), flow rate (L/min)	Pulsatile	Diaphragm / sac-type Pump	Other	Synthetic Tube	No	Multiple repeated experiments per endoleak size (n not explicitly stated)	-

Sidhu et al.	2012	Devices/Products, Radiological	Evaluation of accuracy and efficiency of an electromagnetic 3-dimensional navigation system for endovascular catheterization tasks.	TRE (mm), fluoroscopy time (s), procedure time (s), hits to wall/branch ostia, IC3ST score (1–40)	Pulsatile	Unknown	Glycerol/ glycerin	Phantom (mold)	No	1	-
Sieren et al.	2021	Diagnostics	Evaluation of the accuracy of registration techniques across different vascular imaging modalities for endovascular navigation.	Registration error (mm), alignment deviation (°), processing time (s).	Pulsatile	Diaphragm / sac-type Pump	Glycerol/ glycerin	Phantom (3D printed)	Yes	1	The phantom was composed of a radiopaque skeleton and a 3D vascular model. The skeleton was manufactured using 3D printing that was equipped with radiopaque coating.
Steinlauf et al.	2021	Hemodynamics, Intervention	Comparative assessment of unsteady hemodynamics and perfusion in open, hybrid, and chimney aortic arch repair configurations at varying heart rates.	Velocity (cm/s), shear stress (dyn/cm²), perfusion (L/min), oscillatory shear index (OSI), pulsatility index (PI)	Pulsatile	Piston Pump	Water-based	Glass	No	4	-
Timaran et al.	2004	(Bio)mechanical characteristics, Intervention	Quantification of aneurysm wall pressure relative to endoleak volume in a type II endoleak model.	Aneurysm wall pressure (mmHg), mean arterial pressure (mmHg), endoleak volume (mL), correlation coefficient (r)	Pulsatile	Unknown	Saline-buffered	Phantom (mold)	No	1	-
Tsai et al.	2008	Hemodynamics, No intervention, Pathological	Assessment of the effect of intimal tear size and location on false lumen pressure in chronic type B aortic dissection.	Pressure (mmHg), false lumen pressure index (%), heart rate (BPM), tear diameter (mm)	Pulsatile	Piston Pump	Blood Mimicking	Phantom (mold)	No	3	-
Urbina et al.	2016	Devices/Products, Phantom	Validation of a realistic MRI-compatible aortic phantom for simultaneous flow and cardiac motion studies.	Flow velocity (cm/s), stroke volume (mL), wall displacement (mm), pressure (mmHg).	Pulsatile	Gear Pump	Glycerol/ glycerin	Phantom (mold)	No	1	-
van Disseldorp et al.	2020	Diagnostics	Reproducibility assessment of ultrasound-based PWV and strain measurements in an in-vitro aortic model.	Pulse wave velocity (m/s), strain (%), pressure (mmHg)	Pulsatile	Piston Pump	Saline-buffered	Porcine	No	7	A 3D printed spine and gelatin solution were added to mimic the surrounding tissues in the abdomen
Wang et al.	2018	Diagnostics	Validation of ultrafast ultrasound strain imaging (pStrain) for quantifying aortic aneurysm stiffness in vitro.	Principal strain (%), stiffness (kPa), pressure (mmHg), flow velocity (cm/s).	Pulsatile	Piston Pump	Unknown	Phantom (mold)	No	2; one normal and one aortic aneurym model	-

Wen et al.	2010	(Bio)mechanical characteristics, No intervention, Healthy	Investigation of pulsatile flow fields and wall shear stress in healthy thoracic aorta phantoms using PIV.	WSS (N/m²), OSI, wall pressure (mmHg), velocity (m/s)	Pulsatile	Piston Pump	Glycerol/ glycerin	Phantom (3D printed)	Yes	1	-
Williamson et al.	2021	Hemodynamics, Intervention	Identification of flow disturbances and Type 1B endoleak formation distal to the Frozen Elephant Trunk stent surrogate using PIV.	Velocity (m/s), pressure (mmHg), recirculation zone length (mm).	Pulsatile	Piston Pump	Glycerol/ glycerin	Phantom (3D printed)	No	1	-
Xu et al.	2013	Diagnostics	Feasibility of MR elastography for noninvasive assessment of aortic wall stiffness.	Young's modulus–wall thickness product (E*t, kPa·mm), static pressure (mmHg), wavelength (mm).	Pulsatile and Static	Unknown	Saline-buffered	Porcine	No	1	The phantom was embedded in a gel to mimic tissue: A tissue-simulating 10% B-gel (Bovine Skin Powder) was made in a cylindrical container to mimic the abdominal tissue the mechanical vibrations must pass through to get to the aorta in vivo.
Yazdi et al.	2021	Hemodynamics, No intervention, Healthy	Comparison of hemodynamic flow patterns in compliant versus rigid ascending aorta phantoms under pulsatile flow.	Flow velocity (m/s), shear rate (1/s), wall displacement (mm), diameter change (%).	Pulsatile	Piston Pump	Glycerol/ glycerin	Phantom (mold)	No	2 (1 rigid, 1 compliant)	-
Yusefi et al.	2025	(Bio)mechanical characteristics, Intervention	Comparison of hemodynamic effects between TEVAR and open aortic arch replacement in perfused human thoracic aortas.	Pulse wave velocity (m/s), pressure (mmHg), flow rate (L/min), strain (%).	Pulsatile	Piston Pump	Glycerol/ glycerin	Human	No	11 (TEVAR n=5, OAR n=6)	The aorta was immersed in a liquid bath of 37◦C, preserving its natural curved configuration. Both the circulation and test unit media were a blood-like solution of glycerol-water.
Zehtabi et al.	2023	Devices/Products, Surgical	Evaluation of shear-thinning hydrogel formulations for endoleak embolization.	Viscosity (Pa·s), elastic modulus (G′, Pa), degradation rate (%), flow rate (mL/min).	Pulsatile	Piston Pump	Glycerol/ glycerin	Phantom (3D printed)	No	n=10 per hydrogel group (phantom embolization)	-
Zhang et al.	2016	Diagnostics	Measurement of regional aortic stiffness using MR elastography in phantoms and ex vivo porcine aortas.	Shear stiffness (μMRE, kPa), Young's modulus (kPa), wavelength (mm), pressure (mmHg).	Continu ous	Unknown	Water-based	Phantom + porcine	No	Phantom: 1 setup; Porcine: 4 aortas	The phantom was embedded in a gel to mimic tissue: A tissue-simulating 10% B-gel (Bovine Skin Powder) was made in a cylindrical

											container to mimic the abdominal tissue the mechanical vibrations must pass through to get to the aorta in vivo.
Zimmermann et al.	2021	(Bio)mechanical characteristics, No intervention, Healthy	Assessment of the impact of vessel wall stiffness on flow dynamics and pulse wave velocity in a 3-dimensional printed thoracic aorta model using 4-dimensional flow MRI.	Pulse wave velocity (m/s), pressure (mmHg), flow velocity (cm/s), Young's modulus (MPa).	Pulsatile	Gear Pump	Glycerol/ glycerin	Phantom (3D printed)	Yes	3	-
Zimpfer et al.	2011	Devices/Products, Surgical	Feasibility of stent-graft placement for ascending aortic dissection in a porcine in vitro model.	Pressure (mmHg), true lumen diameter (cm), true lumen area (cm^2), circumference (cm), flow rate (L/min).	Pulsatile	Piston Pump	Water-based	Porcine	No	12	-
